# The genetic risk factors for cerebral venous thrombosis: a case-control study in a Chinese national comprehensive hospital

**DOI:** 10.1186/s12959-024-00621-8

**Published:** 2024-06-17

**Authors:** Shaoying Wang, Ming Yao, Xinzhuang Yang, Yicheng Zhu, Bin Peng

**Affiliations:** 1 Department of Neurology, State Key Laboratory of Complex Severe and Rare Diseases, Peking Union Medical College Hospital, Peking Union Medical College & Chinese Academy of Medical Sciences, Beijing, 100730 China; 2 Center for bioinformatics, National Infrastructures for Translational Medicine, Institute of Clinical Medicine & Peking Union Medical College Hospital, Chinese Academy of Medical Sciences and Peking Union Medical College, Beiing, China

**Keywords:** Cerebral venous thrombosis, Whole exome sequencing, Risk factor, Hereditary thrombophilia, MAP kinase signaling pathway

## Abstract

**Background:**

About 13–25% of cerebral venous thrombosis (CVT) cases lack clear etiology, which may be associated with underlying genetic factors. This study aims to investigate genetic factors in CVT patients using whole exome sequencing (WES).

**Methods:**

Thirty-eight CVT patients hospitalized underwent WES. 977 subjects with WES data from a community cohort study --the Shunyi cohort were as the control group. Using bioinformatics analysis, differential genes with rare damaging variants between two groups were filtered (*P* < 0.05). KEGG enrichment analysis was performed on the screened genes to identify pathways associated with CVT.

**Results:**

Through analysis of medical history, routine tests, and imaging examinations, the etiology of 38 patients: 8 cases of antiphospholipid syndrome, 6 cases with hematologic diseases, 3 cases of protein C deficiency, and 2 cases of protein S deficiency. Five cases occurred during pregnancy or puerperium, and 3 cases had a history of oral contraceptive use, and so on. The etiology was unknown in 12 cases (31.6%), and the etiology of 4 patients were further clarified through WES: F9 c.838 + 1_838 + 16del, Hemizygote: F9 EX1-EX7 Dup; CBS c.430G > A, CBS c.949 A > G; F2 c.1787G > A; SERPINC1 c.409-11G > T. Comparing the WES data of two groups, a total of 179 different genes with rare damaging variants were screened (*P* < 0.05), with 5 genes of interest (JAK2, C3, PROC, PROZ, SERPIND1). Enrichment analysis of the 179 different genes revealed the complement and coagulation pathway and the mitogen activated protein kinases (MAPK) pathway were associated with CVT.

**Conclusion:**

For CVT patients with unknown etiology, WES could help identify the cause of CVT early, which is of great significance for treatment decisions and prognosis. In addition to the complement and coagulation pathway, MAPK pathway is associated with CVT, potentially related to platelet regulation and inflammatory response.

**Supplementary Information:**

The online version contains supplementary material available at 10.1186/s12959-024-00621-8.

## Introduction

Cerebral venous thrombosis (CVT) is a special type of cerebrovascular disease that results from impaired blood flow or cerebrospinal fluid circulation, leading to intracranial hypertension and focal brain damage. The incidence of CVT is approximately 5 per million, accounting for approximately 0.5-3% of cerebrovascular diseases [1, 2].

The pathogenesis of CVT remains incompletely elucidated, intricately linked with diverse risk factors categorized as transient or permanent. Transient risk factors including gender-specific factors like oral contraceptives, pregnancy, puerperium, and hormone replacement therapy, alongside procedural factors such as lumbar punctures and neurosurgical interventions. Additionally, cranial or cervical infections, anemia, and dehydration contribute to transient ones. On the other hand, permanent risk factors involve genetic predispositions to thrombosis, notably prothrombin G20210A mutation, Factor V Leiden mutation (FVL), deficiencies in proteins C (PC), protein S (PS), and antithrombin (AT), Janus Kinase-2 V617F mutation (JAK2 V617F), and MTHFR (C677T) polymorphism [3]. Furthermore, systemic diseases including autoimmune diseases, neoplasms, and myeloproliferative disorders, arteriovenous fistulas, and obesity constitute permanent ones. Hereditary thrombophilia notably contributes to CVT risk, accounting for approximately 34-41% of cases [1, 4]. CVT patients with hereditary thrombophilia may necessitate prolonged or lifelong anticoagulation post-acute phase management.

In addition to the well-established hereditary thrombophilia mentioned above, retrospective case-control investigations have revealed other genetic predispositions to thrombosis such as LDLR mutation, protein Z (G79A), plasma glutathione peroxidase (GPx-3) gene promoter, and XII factor (C46T) gene polymorphisms [5–8]. However, these studies, predominantly relied on candidate genes, which hindered the discovery of new risk genes. Moreover, 13–25% of CVT cases still lack clear risk factors or etiologies, possibly related to underlying undetermined genetic factors [9]. Addressing this gap, the International Stroke Genetics Consortium (ISGC) has been conducting global multicenter studies towards the genetic basis of CVT (the BEAST Consortium) [9]. Given the racial and regional disparities in hereditary factors associated with CVT [4, 10], it is necessary to investigate genetic risk factors among Chinese CVT patients.

Genome-Wide Association Studies (GWAS) focus on common variations. Compared to rare variations, common variations often have smaller effects and are difficult to map to causal genes. Protein-coding genes constitute only approximately 1% of the human genome but harbor 85% of the mutations with significant impacts on disease-related traits [11]. Therefore, selectively sequencing the complete coding regions (i.e., “whole exome”) may potentially contribute to understanding both rare and common human diseases. Whole exome sequencing (WES) enables the study of associations between rare pathogenic and susceptible genetic variations and complex diseases. This study aims to investigate genetic factors in CVT patients using WES in a comprehensive hospital in China.

## Methods

### Study design and patient selection

From June 2017 to June 2023, 38 inpatients diagnosed with CVT were prospectively enrolled consecutively at the Department of Neurology, Peking Union Medical College Hospital (PUMCH). In the study, CVT was diagnosed according to established criteria and confirmed by at least one of the following neuroradiological examinations: computed tomography venography (CTV), magnetic resonance imaging (MRI), magnetic resonance venography (MRV), or digital subtraction angiography (DSA) [12]. Demographic characteristics, medical history, clinical manifestations, laboratory tests, imaging data (CT, MRI, MRV, DSA), and treatment were collected. Laboratory results including thrombophilia tests such as PC, PS, ATIII and activated PC resistance (APC-R), antiphospholipid antibodies (aPL) such as anticardiolipin antibodies (aCL), anti-β2-glycoprotein I antibodies (aB2GPI), lupus anticoagulant, and antinuclear antibodies, and homocysteine. All 38 patients signed informed consent forms for enrollment, and whole blood samples were collected for WES. Severity of illness at admission and discharge was assessed by modified Rankin Scale (mRS) [13].

All controls were recruited from the Shunyi Study, an ongoing prospective population-based cohort aimed at exploring the risk factors and consequences of brain changes in Chinese community-dwelling adults. A detailed recruitment procedure has been previously published [14]. Briefly, all residents aged 35 and above, living independently in five villages in Shunyi, a suburb northeast of Beijing, were invited to participate between June 2013 and April 2016 and a total of 1586 individuals participated in the study. The control group consisted of 977 participants, who had no history of stroke or dementia, and their blood samples were collected for WES.

The study was approved by the local hospital Ethics Committee (JS-2420). Written and informed consent was obtained from all participants.

### Whole exome sequencing

Patients: Blood samples of 38 patients were subjected to high depth WES performed at BGI Genomics Co., Ltd. Genomic DNA was extracted from the blood samples (MagPure Buffy Coat DNA Midi KF Kit, MAGEN). Firstly, the genomic DNA was broken into 100–500 bp fragments by enzyme kit (Shearing Enzyme premix reagent, ENZYMATICS), then the 200–300 bp fragments were collected by magnetic bead (Vahtstm DNA Clean Beads, VAZYME) and prepared into libraries. Subsequently, the KAPA Hyper Exome, ROCHE was employed to capture and enrich the DNA of target gene exons and adjacent splice regions. Finally, mutation detection was conducted using the MGISEQ-2000 sequencing platform. Quality control metrics for sequencing data included: average sequencing depth of the target region ≥ 180X, with over 95% of the sites in the target region having an average depth > 20X.

Data Analysis: The “clean reads” (with a length of 90 bp) derived from targeted sequencing and filtering were then aligned to the human genome reference (hg19) using the BWA (Burrows Wheeler Aligner) Multi-Vision software package [15]. After alignment, the output files were used to perform sequencing coverage and depth analysis of the target region, single-nucleotide variants (SNVs) and INDEL calling. We used GATK software [16] to detect SNVs and indels. All SNVs and indels were filtered and estimated via multiple databases, including NCBI dbSNP, HapMap, 1000 human genome dataset and database of 100 Chinese healthy adults. To predict the effect of missense variants, we used dbNSFP [17] which contains seven well-established in silico prediction programs (Scale-Invariant Feature Transform (SIFT), Polyphen2, LRT, Mutation Taster, and PhyloP). Pathogenic variants were assessed under the protocol issued by ACMG [18]. The Human Gene Mutation Database (HGMD) was used to screen mutations reported in published studies.

Controls: DNA samples of 977 participants were subjected to high depth WES performed at Novogene Corporation (Beijing, China). Detailed recruitment procedure has been previously published [14].

### Variant annotation and definition of rare damaging variants

Variants were further annotated by Variant Effect Predictor (VEP, v105) and the LOFTEE plugin. LOFTEE applies a range of filters on variants annotated as stop-gained, splice site, and frameshift variants to remove those that are unlikely to be disruptive. Those variants labeled as “high-confidence” by LOFTEE were retained. Besides, variants annotated as missense variants were then filtered by (1) CADD score > = 20, or (2) SIFT4G score < 0.05, or (3) PolyPhen-2 score > 0.8. Variants were classified as probably deleterious if they were satisfied criteria in at least 2 software tools. Rare variants were defined using a minor allele frequency (MAF) threshold of 0.1% in all gnomAD database, gnomAD exome database and ExAC East Asian.

Screen out rare damaging variants in the WES data of the case and the control group from a total of 16,317 genes. The number of individuals with rare damaging variants in each gene in the case and control groups was obtained.

### Statistical analysis

Descriptive analyses were conducted for the patients’ characteristics, represented as mean (standard deviation) for continuous variables and frequency (%) for categorical variables. The differences in rare damaging variants between CVT patients and the control group were compared using the Chi-square test. Statistical significance was defined as two-tailed *P*<0.05, unless otherwise specified. Statistical analyses described above were performed using SPSS, version 23.0. To explore the potential function of target genes, Kyoto Encyclopedia of Genes and Genomes (KEGG) enrichment analysis was performed using GeneCodis (http://genecodis.genyo.es/?tdsourcetag=s_pcqq_aiomsg). The cut-off value for significance was *P*<0.05.

## Results

### Characteristics of the CVT patients

Table 1 presented the demographic, clinical, and imaging characteristics of 38 CVT patients. The average age was 39.61 ± 17.20 years, with 20 females and 18 males. Headache was the most common symptom (76.3%), followed by visual disturbances (34.2%), focal neurological deficits (34.2%), altered consciousness (31.6%), seizures (21.1%), and cognitive impairment (21.1%). Tinnitus and abnormal mental behavior were also reported by some patients. The most frequently involved venous sinus was the transverse sinus (57.9%), followed by the sigmoid sinus (55.3%) and the superior sagittal sinus (42.1%). Cortical vein thrombosis was observed in nine patients (23.7%). Additionally, 6 patients (18.4%) had cerebral deep venous thrombosis involving Gallen vein or internal cerebral veins. Approximately 70% of patients had multiple venous sinuses involvement. Eighteen patients presented with hemorrhage, and 21 patients presented with brain swelling and focal edema. Except for 2 patients who died and 1 patient whose condition remained poor, 92.1% of the patient’s showed improvement at discharge. The median mRS at admission was 2, whereas the median mRS at discharge was 1.

**Table 1:** Demographic and clinical characteristics of total patients

	Total patients (*n* = 38)	Percentage (%)
Age (mean ± SD)	39.61 ± 17.20	
Gender		
Male	18	47.4
Female	20	52.6
Clinical characteristics		
Headache	29	76.3
Altered consciousness	12	31.6
Seizure	8	21.1
Visual impairment	13	34.2
Focal neurological deficits	13	34.2
Cognitive impairment	8	21.1
Mental abnormality	2	5.3
Tinnitus	7	18.4
Occluded sinus/vein		
superior sagittal sinus	16	42.1
Transvers sinus	22	57.9
Sigmoid sinus	21	55.3
Inferior sagittal sinus	1	2.6
Straight sinus	7	18.4
Cortical vein	9	23.7
Deep system (VG/ICV)	6	15.8
Internal jugular vein	5	13.2
More than one sinus	27	71.1
Parenchymal lesion on imaging		
Hemorrhagic lesion (parenchymal hemorrhage or subarachnoid hemorrhage)	18	47.4
Brain swelling, focal edema (vasogenic edema or cytotoxic edema)	21	55.3
Anticoagulation treatment	36	94.7
mRS at discharge		
0–2	22	57.9
3–5	16	42.1
6	0	0
mRS at admission		
0–2	32	84.2
3–5	4	10.5
6	2	5.3

*Abbreviation* SD, standard deviation; VG, vein of Galen; ICV, internal cerebral vein

Summarized the classification of risk factors and etiology for 38 cases by analyzing medical history, routine clinical tests, and imaging examinations (Table 2**)**. Among them, 8 patients were diagnosed with antiphospholipid antibody syndrome (APS) (21.1%). Six patients presented concomitant hematologic disorders (15.8%), including thrombocytosis, myelodysplastic syndrome, paroxysmal nocturnal hemoglobinuria (PNH), aplastic anemia, and iron deficiency anemia. Three patients had protein C deficiency, while two others had protein S deficiency. Among the 20 female patients, 5 suffered from CVT during pregnancy or puerperium (25.0%), and 3 had a history of oral contraceptive use (15.0%). In addition, one patient was diagnosed with cystathionine beta-synthase deficiency, another with hypertrophic duraitis, one with concomitant choriocarcinoma, and one with intracranial infection. Eleven patients were found to have hyperhomocysteinemia (hhcy) (30.6%). Nonetheless, the etiology remained unclear in 12 patients (31.6%).

**Table 2:** The classification of risk factors and etiology for 38 patients

Risk factors	*n*/*N*		Percentage (%)
Autoimmune diseases	8/38		21.1
Antiphospholipid antibody syndrome	8		
Hematological disorders	6/38		15.8
Thrombocytosis	2		
Myelodysplastic syndrome	1		
Paroxysmal nocturnal hemoglobinuria	1		
Aplastic anemia	1		
Iron deficiency anemia	1		
Protein S deficiency	2/38		5.3
Protein C deficiency	3/38		7.9
Pregnancy or puerperium	5/20		25.0
Oral contraceptive use	3/20		15.0
Cancer	1/38		2.6
Intracranial infection	1/38		2.6
Other	2/28		5.3
Cystathionine beta-synthase deficiency	1		
Hypertrophic duraitis	1		
Hyperhomocysteinemia	11/36		30.6
Unclear	12/38		31.6

Notably, all 8 patients suspected APS during hospitalization underwent follow-up and obtained repetition of serology at least 12 weeks apart, confirming the diagnosis of APS, with one patient being seronegative APS. Among the 5 patients with PC deficiency or PS deficiency, except for one patient who did not undergo follow-up examinations (Table 3, P1), the remaining 4 patients (Table 3, P 2–5) all underwent follow-up examinations during the non-acute phase, and the results remained abnormal.

**Table 3:** The summarization of etiology for 38 patients combined whole exome sequencing analysis

	Age/Gender	Risk factors/etiology	Homocysteine MTHFR	WES	ACMG
1	40/M	PS deficiency	***38.9*** *MTHFR (TT)*	***PROS1 c.602-1G > T***	VUS
2	43/M	PC deficiency	10.7	***PROC c.565 C > T (p. Arg189Trp)***	VUS
3	26/F	OC use, PC deficiency	10.1 *MTHFR (TT)*	***PROC c.373G > C (p. Gly125Arg)***	VUS
4	26/F	Pregnancy, PC deficiency	10	***PROC c.1218G > A (p. Met406Ile)*** F5 c.3331G > A (p. Ala1111Thr)	Pathogenic VUS
5	35/F	Puerperium, PS deficiency	10.8	***PROS1 c.301 C > T (p. Arg101Cys)*** JAK2 c.2959G > A (p. Glu987Lys)	VUS VUS
6	32/F	Puerperium, APS	7.1	***F8 c.144–1259 C > T*** ***F8 c.6724G > A (p. Val2242Met)***	VUS VUS
7	34/F	Puerperium, APS	13.6 *MTHFR (TT)*	F13B c.986–15 A > G	VUS
8	32/F	Puerperium, APS	8.9 *MTHFR (TT)*	F13A1 c.1081G > A (p. Val361Met)	VUS
9	39/F	APS	9.3	/	/
10	34/M	APS	13.9	/	/
11	32/M	APS	***15.1***	SERPIND1 c.1027G > T (p. Asp343Tyr)	VUS
12	16/F	APS	13.2	/	/
13	32/F	APS	11	/	/
14	83/M	Myelodysplastic syndrome	9.9	F5 c.2032 A > G (p. Lys 678Glu)	VUS
15	70/M	Thrombocytosis	13.8	***JAK2 c.1849G > T (p. Val617Phe)***	Likely pathogenic
16	52/F	Thrombocytosis	***15.1***	***JAK2 c.1849G > T (p. Val617Phe)*** JAK2 c.840 A > G (p. (Ser280=))	Likely pathogenic VUS
17	29/F	Paroxysmal nocturnal hemoglobinuria	***17.3***	***HBB c.79G > A (p. Glu27Lys)*** ***PIGA c.981 + 1G > A***	Pathogenic Pathogenic
18	33/F	Aplastic anemia	10.4	/	/
19	35/F	Iron deficiency anemia	11.4	/	/
20	22/M	FIX↑	***22.9*** *MTHFR (TT)*	***Hemizygote: F9 EX1-EX8E Dup***	VUS
21	35/F	Hormone replacement therapy	7.9	JAK2 c.3177G > A (p. (Ala1059=))	VUS
22	29/F	OC use	12.3	F8 c.4800 A > C (p. Lys1600Asn)	VUS
23	38/F	OC use	N	/	/
24	62/M	Hypertrophic duraitis	14.8	/	/
25	61/M	Intracranial infection	13.8 *MTHFR (TT)*	/	/
26	32/F	Choriocarcinoma	7.5	PROC c.541T > G (p. Phe181Val)	Pathogenic
27	72/M	*Unclear*	***22.4***	F13A1 c.1893T > C (p. (Pro631=))	VUS
28	19/M	*Unclear*	***20.7***	***F9 c.838 + 1_838 + 16del*** ***Hemizygote: F9 EX1-EX7 Dup*** F5 c.2032 A > G (p. Lys678Glu)	Likely pathogenic VUS VUS
29	86/M	*Unclear*	***39.4*** *MTHFR (TT)*	PROS1 c.-190 C > G	VUS
30	40/F	*Unclear*	NA	/	/
31	31/M	*Unclear*	***85.1***	PROC c.577_579delAAG (p. Lys193del)	VUS
32	36/M	*Unclear*	***15.6*** *MTHFR (TT)*	/	/
33	15/M	*Unclear*	***>250***	***CBS c.430G > A (p. Glu144Lys)*** ***CBS c.949 A > G (p. Arg317Gly)***	Likely pathogenic Likely pathogenic
34	61/F	*Unclear*	9.8	/	/
35	49/M	*Unclear*	11.6	***F2 c.1787G > A (p. Arg596Gln)*** PROS1 c.-190 C > G	Pathogenic VUS
36	31/M	*Unclear*	***35.3*** *MTHFR (TT)*	***SERPINC1 c.409-11G > T***	VUS
37	35/F	*Unclear*	8.8	/	/
38	28/M	*Unclear*	10.3	F2 c.1299G > T (p. Arg433Ser)	VUS

*Abbreviation* APS, antiphospholipid antibody syndrome; OC, Oral contraceptive; WES, whole exome sequencing; F, female; M, male; VUS, Variant of Uncertain Significance

The mutations identified by WES, unless otherwise specified, are all heterozygous mutations

### The of etiology for 38 patients combined whole exome sequencing analysis

WES was performed on 38 patients. The primary focus was on gene mutations linked to well-established hereditary thrombophilia, including F2 (prothrombin G20210A mutation), F5 (FVL mutation), PROC mutation, PROS1 mutation, SERPINC1 mutation, JAK2 V617F, and MTHFR (C677T) polymorphism, along with gene mutations associated with the primary diseases or comorbidities. The specific conditions of each CVT patient were detailed in Table 3.

As shown in Table 3, two patients clinically presenting with protein S deficiency were found to have mutations in the PROS1 gene (P1, P5), and P5 suffered from CVT during puerperium. Three patients clinically presenting with protein C deficiency were found to have mutations in the PROC gene (P2, P3, P4). Among them, P3 also had a personal history of oral contraceptive use, and P4 suffered from CVT during pregnancy. For the five patients whose onset occurred during pregnancy or the puerperium, all had additional risk factors or causes. Among them, the two patients with low PS level or PC level both underwent follow-up examinations (P4, P5), and the results were still abnormal (PS 28%→47% (normal range 76–135%); PC 38%→35% (normal range 70–140%)), which were consistent with the gene mutations identified by WES. Except for the two combined hereditary protein S deficiency or hereditary protein C deficiency, the other three patients were diagnosed with APS (P6, P7, P8). P6 also carried a compound heterozygous mutation in the F8 gene. Two patients with combined thrombocytosis were found to have the JAK2 V617F mutation (P15, P16). One patient with PNH was identified with pathogenic mutations in the HBB and PIGA genes through WES (P17). P20 had significantly elevated F9 activity and WES revealed a duplication mutation in the exons of the F9 gene on the X chromosome. In P28, WES also identified a duplication mutation of the F9 gene, along with a deletion mutation; however, FIX activity was not detected in this patient. P35 had a family history of thrombosis (his father suffered from pulmonary embolism), and WES identified a mutation in F2 gene, which was assessed as pathogenic. A total of 12 patients were found to have hhcy, with one patient presenting markedly elevated level of homocysteine (> 250 umol/L) (P33). WES revealed compound heterozygous mutations in the CBS gene in this patient, which can lead to cystathionine beta-synthase deficiency, further predisposing to CVT. Among the remaining 11 patients with hhcy, 5 patients had the MTHFR (TT) polymorphism. Among 12 patients with unclear etiology, the causative etiology of 4 cases was further clarified through the mutation detected by WES. Thirteen patients had multiple risk factors (34.2%).

### Differential genes and underlying pathways associated with CVT from case-control study

**Fig. 1:**
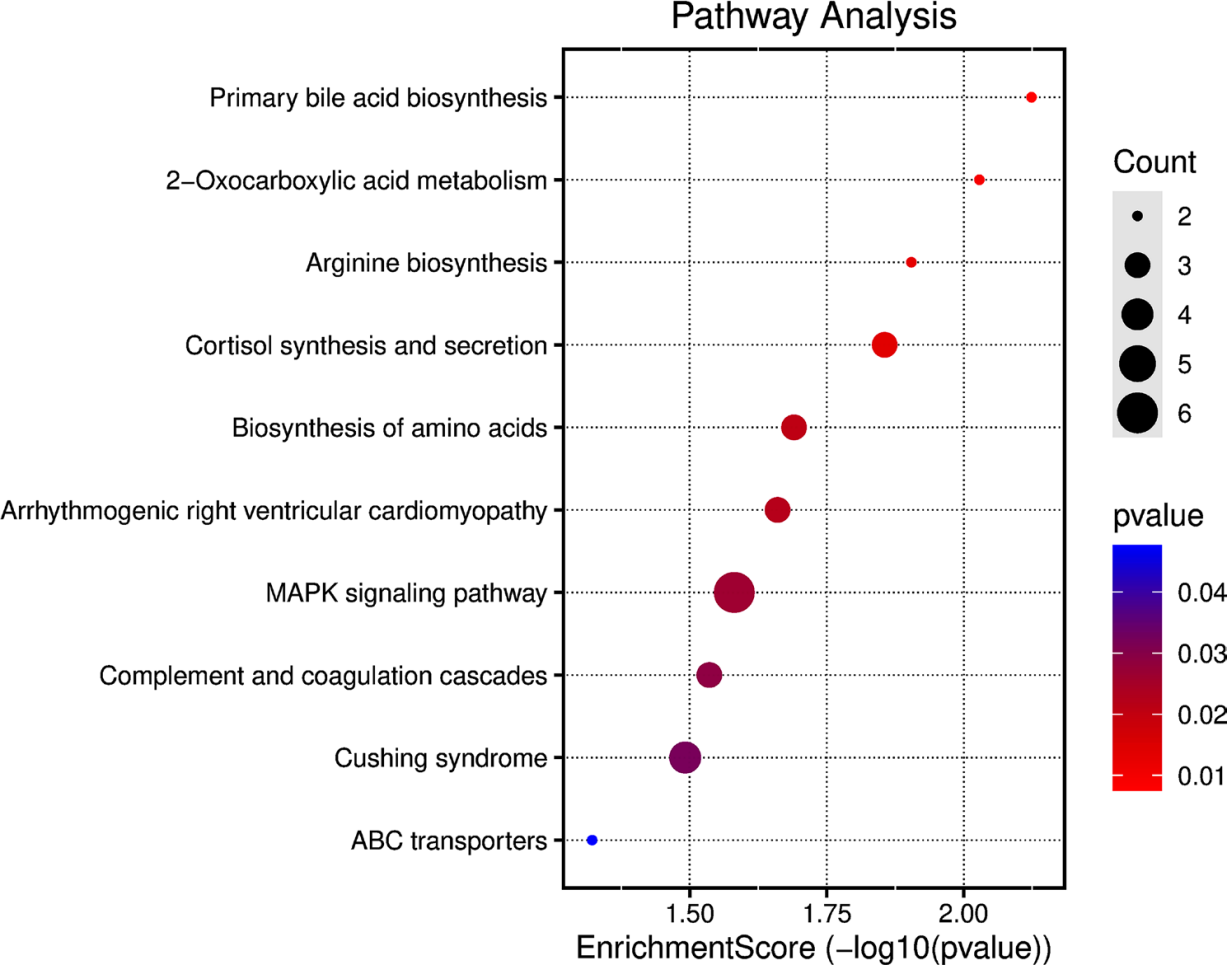
Pathways from enrichment analysis by KEGG

**Fig. 2:**
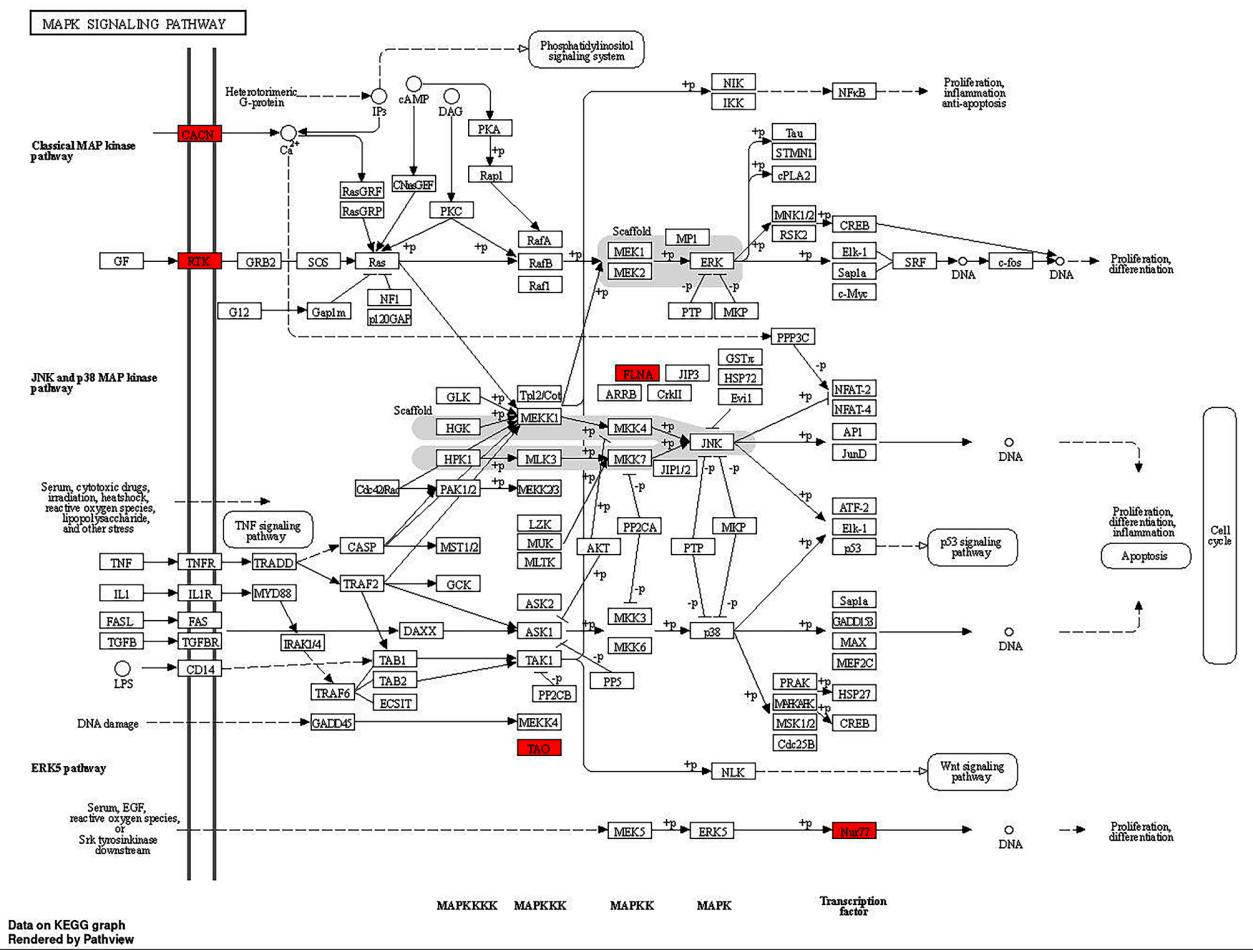
The MAPK signaling pathway in cerebral venous thrombosis enriched by KEGG. Red diamonds represent the enriched target genes’ protein

Drawing from published literature, and genes potentially associated with CVT, such as genes possibly related to APS, we assembled a list of genes of interest (see Supplementary Table 2). To further investigate genes potentially associated with CVT, rare damaging variants with differences between the case and control groups were filtered. A total of 179 genes were screened (see Supplementary Table 3), with 5 genes was in the list of genes of interest (JAK2, C3, PROC, PROZ, and SERPIND1 gene) (Table 4). Enrichment analysis of different 179 genes was conducted using KEGG. Some pathways were obtained and the results are shown in Fig. 1. Among them, the complement and coagulation cascades (involving genes C3, PROC, SERPIND1), and the mitogen activated protein kinases (MAPK) pathway (involving genes FLNB, CACNA1D, TAOK2, CACNB1, FLT4, NR4A) were associated with CVT. Further localization of the genes involved in the MAPK pathway revealed their primary impacts on the specific positions within the MAPK pathway (Fig. 2). Fifteen CVT patients (39.5%) were found to have rare damaging variants in six genes linked to the MAPK pathway, of which two patients lacked identifiable etiology for CVT, while the remaining thirteen patients had known causes or risk factors.

**Table 4:** Differential genes with rare damaging variants in the list of genes of interest

Gene	Case (*n* = 38)		Control (*n* = 977)		*P* value
	Mutation	Un-mutation	Mutation	Un-mutation	
JAK2	3	35	11	966	0.013
C3	2	36	7	970	0.042
PROC	2	36	6	971	0.033
PROZ	2	36	4	973	0.019
SERPIND1	2	36	5	972	0.025

## Discussion

We found that with only medical history, routine laboratory tests, and imaging data, the underlying cause of approximately 30% of CVT patients remained unknown. WES could help identify the cause of CVT early and timely, particularly for patients with unknown cause. By comparing WES data between CVT patients and controls, we identified five genes of interest (JAK2, C3, PROC, PROZ, and SERPIND1 gene) associated with CVT. Enrichment analysis revealed that beyond the complement and coagulation pathway, the MAPK pathway is related to CVT.

Through analyzing the medical history, routine laboratory tests, and imaging examinations of the CVT patients, we summarized the risk factors and etiology. Our findings revealed that autoimmune diseases constituted significant risk factors for CVT, accounting for approximately 21.1% of the CVT patients. A previous study at our hospital identified autoimmune diseases as the predominant cause of CVT cases among women of childbearing age, representing approximately 27.8% [19]. Studies indicated that autoimmune diseases, including Behçet’s syndrome, systemic lupus erythematosus, APS, and Sjögren’s syndrome, were important causes of CVT [20, 21]. In our study, all patients with autoimmune diseases were diagnosed with APS, and 75% of them were female. The mechanism of APS inducing CVT may be theorized that aPL instigate a prothrombotic state by activating endothelial cells, platelets, and monocytes. Furthermore, aB2GPI and complement activation play crucial roles in thrombosis [20, 21]. Additionally, hematologic disorders are important risk factors for CVT. Previous study has reported an association between anemia and CVT [22]. Furthermore, myeloproliferative neoplasms, thrombocytosis, and PNH could also lead to CVT [23–25]. Gender-specific risk factors such as pregnancy, puerperium and oral contraceptive use are important risk factors of CVT [4]. In our study, among 20 female patients, 5 patients suffered from CVT during pregnancy or puerperium, with 3 cases having a history of oral contraceptive use. It is noteworthy that all five pregnancy-related patients had additional risk factors and etiologies, such as APS, PS deficiency, and PC deficiency. This suggested that although pregnancy-induced hypercoagulability increases the risk of CVT, attributing the etiology of CVT solely to pregnancy is overly simplistic and screening for other risk factors should not be overlooked.

Although the etiology of the majority of CVT patients can be clarified through medical history, routine tests, and imaging data, approximately 30% of patients still have unknown causes. The causative etiology of 4 cases was further clarified by WES. WES revealed a duplication and deletion mutation in the F9 gene of patient 28. Patient 20 had similar F9 gene duplication mutation, yet had significantly elevated FIX activity, leading to CVT as detailed in a published article [26]. Additionally, a Turkish study emphasized that F9 gene duplication mutation and increased FIX activity can lead to unique thrombophilia, ultimately resulting in CVT [27]. Patient 33 displayed markedly elevated homocysteine level, and subsequent examinations showed elevated blood methionine levels (approximately double the upper limit of normal) alongside normal urinary methylmalonic acid level. This patient presented bilateral ectopia lentis and a Marfan syndrome-like appearance. Finally, WES identified compound heterozygous mutations in the CBS gene, confirming the diagnosis of cystathionine beta synthase deficiency caused by CBS mutations. The instance of CVT resulting from CBS mutations had been reported [28]. WES identified a mutation in F2 gene in patient 35 (F2 c.1787G > A, Prothrombin Belgrade mutation), which mutation had been confirmed as a pathogenic mutation causing antithrombin resistance [29]. In addition to acute-phase treatments like anticoagulation, dehydration, and life support, patients 33 also received various B-complex vitamins, especially pyridoxine for the primary disease [30]. All four patients underwent long-term anticoagulant therapy and subsequent follow-up showed stable conditions. This further emphasized that for patients with an unknown etiology, WES may assist in promptly identifying the causes of CVT, which could to some extent aid in treatment decision-making and improve patient prognosis. One point to note, in addition to PROC gene mutations, circulating levels of PC may be reduced in several conditions, such as DIC, infection, uremia, cancer or cancer therapy and some autoantibodies [31]. Therefore, in diagnosing hereditary PC deficiency, it is necessary to first rule out the acquired factors. Of course, functional assays of PC take precedence over WES. If several times of functional tests of PC consistently show significant abnormality, validation through WES could be pursued. PS deficiency is similar. Noteworthily, 34.2% patients had multiple risk factors, including genetic and clinical risk factors, and these factors synergistically contributed to the occurrence of CVT. The authors suggest that genetic risk factors may predispose to a potential prothrombotic state leading to CVT, and when combined with certain clinical triggers such as pregnancy or puerperium, oral contraceptives, dehydration, hyperhomocysteinemia, and so on, multiple factors can synergistically reach the threshold for thrombosis.

By screening for rare damaging variants in case and control groups, we identified that the genes JAK2, PROC, PROZ, SERPIND1, and C3 are associated with CVT in the gene of interest. The JAK2 gene, particularly JAK2 V617F mutation, was associated with myeloproliferative neoplasms [32]. Mutations in the PROC gene leading to protein C deficiency, as well as those in the JAK2 gene, had been shown to be specifically associated with CVT [3]. Protein Z is a plasma protein dependent on vitamin K, acting as a cofactor for the protein Z-dependent protease inhibitor to deactivate activated factor X, thereby playing a role in coagulation inhibition. Research on the relationship between the PROZ (G79A) polymorphism and CVT had yielded inconsistent results [5, 33]. Furthermore, the above-mentioned articles were limited to small-sample case-control studies, focusing solely on the polymorphism of the PROZ gene not rare damaging variants of PROZ. SERPIND1 (heparin cofactor II) is a serine protease inhibitor that selectively inactivates thrombin without affecting other proteases involved in the blood coagulation cascade. A previous study indicated an association between mutations in the SERPIND1 gene and venous thromboembolism [34], yet there is currently no study about SERPIND1 gene and CVT. Evidences had indicated interactions between the hemostatic system and innate immunity, and the coagulation and complement cascades. Complement factors make distinct contributions to platelet activation and fibrin deposition. Deficiency in C3 leads to prolonged bleeding time, with C3 playing a specific role in platelet activation [35]. However, there is currently very limited research on the complement and thrombosis, with no studies regarding the association between C3 with CVT. Our study confirmed the well-established genes such as PROC and JAK2, while also proposing the less-studied genes PROZ, SERPIND1, and C3, thus offering direction and insights for future research.

Enrichment analysis revealed that the complement and coagulation pathway was associated with CVT, which had been well-established. Notably, to the best of our knowledge, our study firstly reported a possible association between the MAPK pathway and CVT. In our study, fifteen CVT patients had rare damaging variants in six genes related to the MAPK pathway. Among them, two patients had no identified cause for CVT, while the other thirteen had known causes or risk factors. This indicated that changes in the MAPK pathway could serve as a potential etiology in certain patients with unexplained CVT, even after excluding known possible causes, and may synergistically contribute to the pathogenesis of CVT in patients with specific etiologies. MAPK is a part of the protein kinase cascade, with three identified families: C-Jun N-terminal kinase (JNK), p38 MAPK, and extracellular signal-regulated kinase (ERK). Each cascade requires at least three enzymes for activation: MAPKK kinase, MAPK kinase, and then to MAPK [36]. Dysregulation of MAPK signaling often leads to cancer, diabetes, and other diseases associated with immune response or inflammation. A few articles also emphasized the role of the MAPK pathway in regulating platelet function and thrombosis. Activation of ERK in platelets is crucial for collagen-induced platelet secretion and aggregation [37], and inhibition of its upstream MEK1/2 can suppress ERK activation and prolong occlusion time of arterial and venous thrombosis in mice [38]. Additionally, activation of p38 in collagen-stimulated platelets leads to increased platelet adhesion and spreading, as well as enhanced thromboxane A2 formation [39]. The absence of JNK in platelets also results in impaired platelet aggregation and granule release upon agonist stimulation, and inhibits vascular thrombosis in mouse cecum [40]. The MAPK-interacting kinase 1 regulate mRNA translation and cellular activation in platelets and megakaryocytes, endomitosis and thrombopoiesis, and thrombosis [41]. Furthermore, inflammation and thrombosis are closely linked processes. The p38 MAPK pathway plays a critical role in thrombin-induced endothelial proinflammatory activation [42]. In APS, a major mechanism of hypercoagulability is mediated by aPL, which upregulate tissue factor on monocytes through the p38 MAPK and NF-κB pathways [43]. A previous study found that MAPK signaling pathway was associated with deep venous thrombosis [44]. However, the MAPK signaling pathway is a vast and complex network, with limited research on its association with venous thrombosis. Further studies are needed to confirm the relationship between the MAPK pathway and CVT.

Our study has several limitations that should be acknowledged. The sample size of patients is small. The relationship between the MAPK pathway and CVT has not been experimentally validated. In addition, recruitment was limited to one hospital by Han Chinese people; therefore, caution is necessary when generalizing our findings to other ethnical groups.

## Conclusion

This prospective case-control study of CVT patients enrolled from a comprehensive hospital in China, suggested that the role of genetic risk factors of CVT is indispensable and for patients with unknown etiology, WES could help identify the cause of CVT timely, which could to some extent aid in treatment decision-making and improve patient prognosis. In addition to the complement and coagulation pathways, we found that the MAPK pathway is associated with CVT, which is mainly related to platelet regulation and inflammation. The specific mechanism awaits further investigation through basic experiments.

### Electronic supplementary material

Below is the link to the electronic supplementary material.

Supplementary Material 1

## Data Availability

The data that support the findings of this study are not openly available due to reasons of sensitivity and are available from the corresponding author upon reasonable request. Data are located in controlled access data storage at Peking Union Medical College Hospital.

## Abbreviations

CVT: Cerebral venous thrombosis
WES: Whole exome sequencing
MAPK: Mitogen activated protein kinases
FVL: Factor V Leiden
PC: Proteins C
PS: Protein S
AT: Antithrombin
JAK2 V617F: Janus Kinase-2 V617F
ISGC: International Stroke Genetics Consortium
GWAS: Genome-Wide Association Studies
CTV: Computed tomography venography
MRI: Magnetic resonance imaging
MRV: Magnetic resonance venography
DSA: Digital subtraction angiography
APC-R: Activated PC resistance
aPL: Antiphospholipid antibodies
aCL: Anticardiolipin antibodies
aB2GPI: Anti-β2-glycoprotein I antibodies
SNVs: Single-nucleotide variants
HGMD: Human Gene Mutation Database
MAF: Minor allele frequency
KEGG: Kyoto Encyclopedia of Genes and Genomes
APS: Antiphospholipid antibody syndrome
hhcy: Hyperhomocysteinemia
PNH: Paroxysmal nocturnal hemoglobinuria
JNK: C-Jun N-terminal kinase
ERK: Extracellular signal-regulated kinase
