# Supplementary material for: The genetic risk factors for cerebral venous thrombosis: a case-control study in a Chinese national comprehensive hospital
Source: Thromb J. 2024 Jun 17;22:50. doi: 10.1186/s12959-024-00621-8 (PMC11181614; doi:10.1186/s12959-024-00621-8)
Supplement: Supplementary file 1 — Supplementary Material 1 [file 12959_2024_621_MOESM1_ESM.docx]

Supplementary Table 1 Gene mutations and their software prediction results

|  | WES | ACMG | SIFT | Mutation taster | Condel | SpliceAI | dbscSNV_ RF | dbscSNV ADA | PhyloP  Vertebrates | PhyloP  Placetal  Mammals | GERP++ |  |
| --- | --- | --- | --- | --- | --- | --- | --- | --- | --- | --- | --- | --- |
| *1* | ***PROS1 c.602-1G>T*** | VUS | - | D | - | D | D | D | C | C | C |  |
| *2* | ***PROC c.565C>T (p. Arg189Trp)*** | VUS | P | P | P | P | - | - | NC | NC | NC |  |
| *3* | ***PROC c.373G>C (p. Gly125Arg)*** | VUS | D | D | D | P | - | - | C | C | C |  |
| *4* | ***PROC c.1218G>A (p. Met406Ile)***  F5 c.3331G>A (p. Ala1111Thr) | Pathogenic  VUS | -  P | D  P | D  - | P  P | -  - | -  - | C  NC | C  NC | C  C |  |
| *5* | ***PROS1 c.301C>T (p. Arg101Cys)***  JAK2 c.2959G>A (p. Glu987Lys) | VUS  VUS | D  D | P  D | D  D | P  P | -  - | -  - | NC  C | NC  C | C  C |  |
| *6* | ***F8 c.144-1259C>T***  ***F8 c.6724G>A (p. Val2242Met)*** | VUS  VUS | -  I | -  D | -  D | P  P | -  D | -  D | NC  NC | NC  C | -  C |  |
| 7 | F13B c.986-15A>G | VUS | - | - | - | D | - | - | NC | NC | - |  |
| 8 | F13A1 c.1081G>A (p. Val361Met) | VUS | P | P | P | P | - | - | NC | NC | C |  |
| 11 | SERPIND1 c.1027G>T (p. Asp343Tyr) | VUS | D | D | D | P | - | - | C | C | C |  |
| 14 | F5 c.2032A>G (p. Lys 678Glu) | VUS | D | P | - | P | - | - | NC | NC | NC |  |
| *15* | ***JAK2 c.1849G>T (p. Val617Phe)*** | Likely  pathogenic | D | D | D | P | - | - | C | C | C |  |
| *16* | ***JAK2 c.1849G>T (p. Val617Phe)***  JAK2 c.840A>G (p. (Ser280=)) | Likely  pathogenic VUS | D  - | D  - | D  - | P  D | -  - | -  - | C  NC | C  C | C  - |  |
| *17* | ***HBB c.79G>A (p. Glu27Lys)***  ***PIGA c.981+1G>A*** | Pathogenic  Pathogenic | D  - | D  D | D  - | D  D | -  D | -  D | NC  C | NC  C | C  C |  |
| *20* | ***Hemizygote F9 EX1-EX8E Dup*** | VUS | - | - | - | - | - | - | - | - | - |  |
| 21 | JAK2 c.3177G>A (p. (Ala1059=)) | VUS | - | - | - | D | D | D | NC | NC | - |  |
| 22 | F8 c.4800A>C (p. Lys1600Asn) | VUS | P | P | P | P | - | - | NC | NC | NC |  |
| 26 | PROC c.541T>G (p. Phe181Val) | Pathogenic | D | D | P | D | - | - | C | NC | C |  |
| 27 | F13A1 c.1893T>C (p. (Pro631=)) | VUS | - | - | - | P | - | - | NC | NC | - |  |
| *28* | ***F9 c.838+1_838+16del***  ***Hemizygote F9 EX1-EX7 Dup***  ***F5 c.2032A>G (p. Lys678Glu)*** | Likely  pathogenic  VUS  VUS | -  -  D | -  -  P | -  -  - | -  -  P | -  -  - | -  -  - | -  -  NC | -  -  NC | -  -  NC |  |
| 29 | PROS1 c.-190C>G | VUS | - | - | - | P | - | - | NC | NC | - |  |
| 31 | PROC c.577_579delAAG (p. Lys193del) | VUS | - | - | - | P | - | - | - | - | - |  |
| *34* | ***CBS c.430G>A (p. Glu144Lys)***  ***CBSc.949A>G (p. Arg317Gly)*** | Likely  pathogenic  Likely  pathogenic | D  D | D  D | D  D | P  P | -  - | -  - | C  NC | NC  NC | C  NC |  |
| *35* | ***F2 c.1787G>A (p. Arg596Gln)***  PROS1 c.-190C>G | Pathogenic  VUS | -  - | D  - | D  - | P  P | -  - | -  - | C  NC | C  NC | C  - |  |
| *36* | ***SERPINC1 c.409-11G>T*** | VUS | D | P | D | P | D | D | C | C | - |  |
| 38 | F2 c.1299G>T (p. Arg433Ser) | VUS | - | D | P | P | D | D | NC | NC | C |  |

Abbreviation: VUS, Variant of Uncertain Significance; Deleterious, D; Polymorphism, P; Conservation, C; Not Conservation, NC; Inconsistency, I.

The mutations identified by WES, unless otherwise specified, are all heterozygous mutations.

Supplementary Table 2 List of genes of interest drawing from literatures

| ***Genes with established roles in coagulation*** | |
| --- | --- |
| CPB2 | Thrombin-Activatable Fibrinolysis Inhibitor |
| FII | Coagulation Factor ll; prothrombin |
| TF(FIII) | Tissue Factor |
| FV | Coagulation Factor V |
| FVll | Coagulation Factor VIl |
| FVIlI | Coagulation Factor VIll |
| FIX | Coagulation Factor IX |
| FX | Coagulation Factor X |
| FXI | Coagulation Factor Xl |
| FXII | Coagulation Factor XIl |
| FXIII | Coagulation Factor XIll |
| FGA | Fibrinogen, A Alpha Polypeptide |
| FGB | Fibrinogen, B Beta Polypeptide |
| FGG | Fibrinogen, G Gamma Polypeptide |
| HABP2 | Factor Vll-Activating Protease |
| HRG | Histidine-Rich Glycoprotein |
| PLAT | Tissue Plasminogen Activator |
| PLAU | Urinary Plasminogen Activator |
| PLG | Plasminogen |
| PROC | Protein C |
| PROCR | Protein C Receptor |
| PROS1 | Protein S |
| SERPINA10(ZPI) | Protein Z-dependent Protease Inhibitor |
| SERPINC1 | Antithrombin |
| SERPIND1 | Heparin Cofactor Il |
| SERPINE1 | Plasminogen Activator Inhibitor-1 |
| SERPINF2 | Alpha-2-Antiplasmin |
| THBD | Thrombomodulin |
| TFPI | Tissue Factor Pathway Inhibitor |
| vWF | von Willebrand Factor |
| ***Genes with roles in platelet function*** |  |
| CALR | Calreticulin |
| GP6 | Platelet Glycoprotein VI |
| JAK2 | Janus Kinase 2 |
| MPL | Thrombopoietin Receptor |
| ***Genes associated with low protein C*** |  |
| BAZ1B | Bromodomain Adjacent to Zinc Finger Domain, 1B |
| CADM1 | Immunoglobulin Superfamily, Member 4 |
| GCKR | Glucokinase Regulatory Protein |
| EDEM2 | Endoplasmic Reticulum Degradation-Enhancing Apha-Mannosidase-Like Protein 2 |
| ***Genes associated with elevated vWF and/or factor Vlll levels*** | |
| ADAMTS13 | von Willebrand Factor-Cleaving Protease |
| ABO | ABO Glycosyltransferase |
| STXBP5 | Syntaxin-Binding Protein 5 |
| TC2N | Transcobalamin ll |
| ***Genes without established roles in coagulation*** | |
| ACE | Angiotensin I-Converting Enzyme |
| C4BPA | C4b Receptor |
| CYP4V2 | Cytochrome P450, Family 4, Subfamily V, Polypeptide 2 |
| HIVEP1 | Human lmmunodeficiency Virus Type 1 Enhancer-Binding Protein 1 |
| KLKB1 | Prekallikrein |
| KNG1 | Kininogen 1 |
| NAT8B | N-Acetyltransferase 8B |
| NR112 | Pregnane X Receptor |
| RGS7 | Regulator of G Protein Signaling 7 |
| SH2B3(LNK) | SH2B Adaptor Protein 3 |
| SLC44A2 | Choline Transporter-Like Protein 2 |
| STAB2 | Stabilin 2 |
| TSPAN15 | Tetraspanin 15 |
| ***Genes associated with APS*** |  |
| CFH | Complement Factor H |
| CFHR1 | Complement Factor H Related 1 |
| CFHR2 | Complement Factor H Related 2 |
| CFHR3 | Complement Factor H Related 3 |
| CFHR5 | Complement Factor H Related 5 |
| CFI | Complement Factor I |
| C3 | Complement C3 |
| CFB | Complement Factor B |
| CFD | Complement Factor D |
| CFP | Complement Factor Properdin |
| DGKE | Diacylglycerol Kinase Epsilon |
| STAT4 | signal transducer and activator of transcription-4 |
| BLK | BLK proto-oncogene |
| IRF5 | Interferon regulatory factor5 |
| TSHR | Thyroid Stimulating Hormone Receptor |
| C1D | C1D Nuclear Receptor Corepressor |
| ATXN2 | Ataxin 2 |
| B2GP1 | Beta-2-glycoprotein I |
| NCF1 | Neutrophil Cytosolic Factor 1 |
| CR1 | Complement C3b/C4b Receptor 1 |
| CFHR4 | Complement Factor H Related 4 |
| HLA-DRB1 | Major Histocompatibility Complex, Class II, DR Beta 1 |
| ITGA2 | Integrin subunit alpha 2 |
| ITGB3 | Integrin subunit beta 3 |
| GP1BA | Glycoprotein Ib platelet subunit alpha |
| PTPN22 | Protein tyrosine phosphatase non-receptor type 22 |
| SELP | Selectin P |
| TFPI | Tissue factor pathway inhibitor |
| TLR4 | Toll-like receptor 4 |
| TNF | Tumor necrosis factor |
| TLR2 | Toll-like receptor 2 |
| PF4V1 | Platelet Factor 4 Variant 1 |
| F2RL1 | Coagulation factor II receptor-like 1 |
| VEGFA | Vascular endothelial growth factor A |
| FLT1 | FMS-related tyrosine kinase 1 |
| IKZF1 | IKAROS Family Zinc Finger 1 |
| MTOR | Mechanistic Target Of Rapamycin Kinase |
| CYBA | Cytochrome B-245 Alpha Chain |
| NOS3 | endothelial NO-synthase |
| SELPLG | P-selectin glycoprotein ligand-1 |
| ***Others*** |  |
| MTHFR | Methylenetetrahydrofolate Reductase |
| PROZ | Protein Z, Vitamin K Dependent Plasma Glycoprotein |
| VKORC1 | Vitamin K Epoxide Reductase Complex Subunit 1 |
| CBS | Cystathionine Beta-Synthase |
| PMS2 | PMS1 Homolog 2, Mismatch Repair System Component |
| TP53 | Tumor Protein P53 |
| HBB | Hemoglobin Subunit Beta |
| PIGA | Phosphatidylinositol Glycan Anchor Biosynthesis Class A |
| DKC1 | Dyskerin Pseudouridine Synthase 1 |
| ITGA2B | Integrin Subunit Alpha 2b |
| MMACHC | Metabolism Of Cobalamin Associated C |
| NOTCH3 | Notch Receptor 3 |
| HTRA1 | HtrA Serine Peptidase 1 |
| APOB | Apolipoprotein B |
| LDLR | Low Density Lipoprotein Receptor |
| CTLA4 | Cytotoxic T-Lymphocyte Associated Protein 4 |
| RASGRP2 | RAS Guanyl Releasing Protein 2 |
| PIGT | Phosphatidylinositol Glycan Anchor Biosynthesis Class T |
| CD36 | CD36 Molecule |
| CD55 | CD55 Molecule |
| GGCX | Gamma-Glutamyl Carboxylase |
| COL4A1 | Collagen Type IV Alpha 1 Chain |
| CRP | C-Reactive Protein |
| CYP3A5 | Cytochrome P450 Family 3 Subfamily A Member 5 |
| MAST2 | Microtubule Associated Serine/Threonine Kinase 2 |
| SLC34A1 | Solute Carrier Family 34 Member 1 |
| YPEL4 | Yippee Like 4 |
| CLP1 | Cleavage Factor Polyribonucleotide Kinase Subunit 1 |
| ZDHHC5 | Zinc Finger DHHC-Type Palmitoyltransferase 5 |
| CBX5 | Chromobox 5 |
| LIPC | Lipase C, Hepatic Type |
| SERPINA1 | Serpin Family A Member 1 |
| SERPING1 | Serpin Family G Member 1 |
| THBS1 | Thrombospondin 1 |

Supplementary Table 3 179 genes with rare damaging variants filtered from case and control groups

| Genename | Mutant  in case | Unmutatant  in case | mutant  in control | unmutatant  in control |
| --- | --- | --- | --- | --- |
| DNAH8 | 7 | 33 | 59 | 918 |
| MEGF8 | 6 | 34 | 33 | 944 |
| MPDZ | 6 | 34 | 53 | 924 |
| FLNB | 5 | 35 | 38 | 939 |
| RBP3 | 5 | 35 | 27 | 950 |
| TJP2 | 5 | 35 | 11 | 966 |
| ZFHX3 | 5 | 35 | 41 | 936 |
| ABCB5 | 4 | 36 | 20 | 957 |
| CECR2 | 4 | 36 | 12 | 965 |
| CPAMD8 | 4 | 36 | 19 | 958 |
| HPS5 | 4 | 36 | 14 | 963 |
| OR4A5 | 4 | 36 | 10 | 967 |
| SSC5D | 4 | 36 | 22 | 955 |
| TRIOBP | 4 | 36 | 24 | 953 |
| ABCG1 | 3 | 37 | 12 | 965 |
| ADAMTS5 | 3 | 37 | 12 | 965 |
| ADAMTS9 | 3 | 37 | 13 | 964 |
| AKNA | 3 | 37 | 8 | 969 |
| AOC1 | 3 | 37 | 14 | 963 |
| ARHGEF38 | 3 | 37 | 9 | 968 |
| ARID4B | 3 | 37 | 9 | 968 |
| ATP10A | 3 | 37 | 11 | 966 |
| BBS1 | 3 | 37 | 15 | 962 |
| CACNA1D | 3 | 37 | 17 | 960 |
| DOP1A | 3 | 37 | 10 | 967 |
| FASTKD1 | 3 | 37 | 17 | 960 |
| FRMPD1 | 3 | 37 | 12 | 965 |
| FRMPD2 | 3 | 37 | 12 | 965 |
| HECTD1 | 3 | 37 | 6 | 971 |
| HECW2 | 3 | 37 | 8 | 969 |
| JAK2 | 3 | 37 | 11 | 966 |
| KBTBD13 | 3 | 37 | 12 | 965 |
| MBD6 | 3 | 37 | 15 | 962 |
| NUP188 | 3 | 37 | 15 | 962 |
| PCDHB14 | 3 | 37 | 10 | 967 |
| PDZD7 | 3 | 37 | 12 | 965 |
| PTPN23 | 3 | 37 | 18 | 959 |
| PTPRD | 3 | 37 | 13 | 964 |
| PTPRU | 3 | 37 | 14 | 963 |
| RABL6 | 3 | 37 | 7 | 970 |
| RAP1GAP | 3 | 37 | 11 | 966 |
| SH3TC2 | 3 | 37 | 9 | 968 |
| SHCBP1 | 3 | 37 | 13 | 964 |
| SIPA1L1 | 3 | 37 | 13 | 964 |
| SLC28A1 | 3 | 37 | 12 | 965 |
| SLC38A6 | 3 | 37 | 9 | 968 |
| SLC4A3 | 3 | 37 | 13 | 964 |
| SLFNL1 | 3 | 37 | 6 | 971 |
| SYT12 | 3 | 37 | 6 | 971 |
| TAOK2 | 3 | 37 | 18 | 959 |
| TCHH | 3 | 37 | 10 | 967 |
| THEG | 3 | 37 | 17 | 960 |
| TRABD2A | 3 | 37 | 13 | 964 |
| TTC3 | 3 | 37 | 13 | 964 |
| TXLNB | 3 | 37 | 5 | 972 |
| TYK2 | 3 | 37 | 18 | 959 |
| VPS16 | 3 | 37 | 9 | 968 |
| WDR75 | 3 | 37 | 8 | 969 |
| ZFP90 | 3 | 37 | 1 | 976 |
| ZNF366 | 3 | 37 | 4 | 973 |
| ZNF503 | 3 | 37 | 4 | 973 |
| ZNHIT6 | 3 | 37 | 13 | 964 |
| ABHD14A-ACY1 | 2 | 38 | 5 | 972 |
| ABHD15 | 2 | 38 | 3 | 974 |
| ACY1 | 2 | 38 | 5 | 972 |
| ADGRA1 | 2 | 38 | 3 | 974 |
| ADGRE5 | 2 | 38 | 4 | 973 |
| ADIPOQ | 2 | 38 | 1 | 976 |
| AMACR | 2 | 38 | 3 | 974 |
| ANGPTL7 | 2 | 38 | 4 | 973 |
| ANKRD65 | 2 | 38 | 3 | 974 |
| ASMTL | 2 | 38 | 6 | 971 |
| ATP5F1A | 2 | 38 | 5 | 972 |
| AUTS2 | 2 | 38 | 7 | 970 |
| AZGP1 | 2 | 38 | 2 | 975 |
| B4GALNT1 | 2 | 38 | 5 | 972 |
| BFSP1 | 2 | 38 | 5 | 972 |
| C2orf76 | 2 | 38 | 4 | 973 |
| C3 | 2 | 38 | 7 | 970 |
| CACNB1 | 2 | 38 | 3 | 974 |
| CALHM2 | 2 | 38 | 6 | 971 |
| CARS | 2 | 38 | 7 | 970 |
| CNTN3 | 2 | 38 | 7 | 970 |
| CRTAC1 | 2 | 38 | 7 | 970 |
| CSRNP2 | 2 | 38 | 2 | 975 |
| CYP11B1 | 2 | 38 | 4 | 973 |
| CYP46A1 | 2 | 38 | 5 | 972 |
| DEAF1 | 2 | 38 | 1 | 976 |
| DGKZ | 2 | 38 | 5 | 972 |
| DUS2 | 2 | 38 | 6 | 971 |
| FBXL17 | 2 | 38 | 6 | 971 |
| FEZ1 | 2 | 38 | 4 | 973 |
| FLT4 | 2 | 38 | 4 | 973 |
| GLIPR1L1 | 2 | 38 | 1 | 974 |
| GNA14 | 2 | 38 | 7 | 970 |
| GOLGA5 | 2 | 38 | 2 | 975 |
| GP2 | 2 | 38 | 3 | 974 |
| GPRIN1 | 2 | 38 | 7 | 970 |
| HBQ1 | 2 | 38 | 1 | 974 |
| HEMK1 | 2 | 38 | 4 | 973 |
| HMOX1 | 2 | 38 | 5 | 972 |
| INPPL1 | 2 | 38 | 5 | 972 |
| KAT2A | 2 | 38 | 3 | 974 |
| KCNA10 | 2 | 38 | 5 | 972 |
| KCNAB1 | 2 | 38 | 4 | 973 |
| KHNYN | 2 | 38 | 7 | 970 |
| KLF3 | 2 | 38 | 2 | 975 |
| KRT38 | 2 | 38 | 5 | 972 |
| LMTK3 | 2 | 38 | 5 | 972 |
| LRRTM2 | 2 | 38 | 4 | 973 |
| MAP3K21 | 2 | 38 | 7 | 970 |
| MARCHF10 | 2 | 38 | 7 | 970 |
| MED29 | 2 | 38 | 1 | 976 |
| MESP1 | 2 | 38 | 6 | 971 |
| MMRN2 | 2 | 38 | 6 | 971 |
| MOV10 | 2 | 38 | 6 | 971 |
| MTFP1 | 2 | 38 | 6 | 971 |
| MYOCD | 2 | 38 | 7 | 970 |
| MYRIP | 2 | 38 | 2 | 975 |
| NCLN | 2 | 38 | 7 | 970 |
| NDFIP2 | 2 | 38 | 4 | 973 |
| NEURL2 | 2 | 38 | 2 | 975 |
| NR4A1 | 2 | 38 | 6 | 971 |
| NTHL1 | 2 | 38 | 2 | 975 |
| OR2W3 | 2 | 38 | 4 | 973 |
| OR5L1 | 2 | 38 | 6 | 971 |
| PABPC4L | 2 | 38 | 6 | 971 |
| PAK5 | 2 | 38 | 6 | 971 |
| PDIA6 | 2 | 38 | 4 | 973 |
| PDLIM5 | 2 | 38 | 4 | 973 |
| PDZRN3 | 2 | 38 | 7 | 970 |
| PIBF1 | 2 | 38 | 6 | 971 |
| PIGH | 2 | 38 | 2 | 975 |
| PLEKHB1 | 2 | 38 | 7 | 970 |
| POLDIP2 | 2 | 38 | 3 | 974 |
| PROC | 2 | 38 | 6 | 971 |
| PROZ | 2 | 38 | 4 | 973 |
| PRPS1L1 | 2 | 38 | 4 | 973 |
| PSMB8 | 2 | 38 | 7 | 970 |
| RABEP2 | 2 | 38 | 4 | 973 |
| RAD9A | 2 | 38 | 7 | 970 |
| RAVER1 | 2 | 38 | 6 | 971 |
| RBM26 | 2 | 38 | 4 | 973 |
| RNMT | 2 | 38 | 3 | 974 |
| RSBN1 | 2 | 38 | 6 | 971 |
| SALL1 | 2 | 38 | 7 | 970 |
| SCAF4 | 2 | 38 | 3 | 974 |
| SCCPDH | 2 | 38 | 2 | 975 |
| SCG3 | 2 | 38 | 3 | 974 |
| SERPIND1 | 2 | 38 | 5 | 972 |
| SETD6 | 2 | 38 | 3 | 974 |
| SF3B2 | 2 | 38 | 4 | 973 |
| SLC15A5 | 2 | 38 | 1 | 974 |
| SLC22A2 | 2 | 38 | 4 | 973 |
| SLC43A2 | 2 | 38 | 7 | 970 |
| SMTNL2 | 2 | 38 | 3 | 974 |
| SNW1 | 2 | 38 | 6 | 971 |
| SORD | 2 | 38 | 5 | 972 |
| SPATA16 | 2 | 38 | 4 | 973 |
| SREK1 | 2 | 38 | 6 | 971 |
| SUN2 | 2 | 38 | 7 | 970 |
| SYT4 | 2 | 38 | 2 | 975 |
| SYTL4 | 2 | 38 | 3 | 974 |
| TAS2R43 | 2 | 38 | 1 | 972 |
| TBC1D13 | 2 | 38 | 2 | 975 |
| TBRG1 | 2 | 38 | 7 | 970 |
| TCF7L1 | 2 | 38 | 7 | 970 |
| TGM4 | 2 | 38 | 5 | 972 |
| TRPM3 | 2 | 38 | 6 | 971 |
| TTK | 2 | 38 | 1 | 975 |
| TXNDC16 | 2 | 38 | 6 | 971 |
| WDR19 | 2 | 38 | 7 | 970 |
| ZC3H14 | 2 | 38 | 7 | 970 |
| ZCCHC8 | 2 | 38 | 6 | 971 |
| ZCWPW2 | 2 | 38 | 3 | 974 |
| ZNF622 | 2 | 38 | 3 | 974 |
| ZNF691 | 2 | 38 | 4 | 973 |
| ZNF717 | 2 | 38 | 3 | 974 |
| ZNF850 | 2 | 38 | 2 | 975 |
